# CSF enhancement on post-contrast fluid-attenuated inversion recovery images; a systematic review

**DOI:** 10.1016/j.nicl.2020.102456

**Published:** 2020-10-02

**Authors:** Whitney M. Freeze, Merel van der Thiel, Jeroen de Bresser, Catharina J.M. Klijn, Ellis S. van Etten, Jacobus F.A. Jansen, Louise van der Weerd, Heidi I.L. Jacobs, Walter H. Backes, Susanne J. van Veluw

**Affiliations:** a Department of Radiology, Leiden University Medical Center, Leiden, the Netherlands; b Department of Neuropsychology and Psychiatry, Alzheimer Center Limburg, School for Mental Health and Neuroscience, Maastricht University, Maastricht, the Netherlands; c Department of Radiology and Nuclear Medicine, School for Mental Health and Neuroscience, Maastricht University Medical Center, Maastricht, the Netherlands; d Department of Neurology, Donders Institute for Brain, Cognition and Behaviour, Radboud University Medical Centre, Nijmegen, the Netherlands; e Department of Neurology, Leiden University Medical Center, Leiden, the Netherlands; f Department of Electrical Engineering, Eindhoven University of Technology, Eindhoven, the Netherlands; g Department of Human Genetics, Leiden University Medical Center, Leiden, the Netherlands; h Gordon Center for Medical Imaging, Massachusetts General Hospital, Harvard Medical School, Boston, MA, USA; i Cardiovascular Research Institute Maastricht, Maastricht University Medical Center, Maastricht, the Netherlands; j Department of Neurology, J. Philip Kistler Stroke Research Center, Massachusetts General Hospital and Harvard Medical School, Boston, MA, USA

**Keywords:** Blood–brain barrier, Fluid-attenuated inversion recovery, Gadolinium, Cerebrospinal fluid, Neurological disease

## Abstract

Cerebrospinal fluid (CSF) enhancement on T2-weighted post-contrast fluid-attenuated inversion recovery (pcT2wFLAIR) images is a relatively unknown neuroradiological marker for gadolinium-based contrast agent extravasation due to blood–brain barrier (BBB) disruption. We systematically reviewed human studies reporting on CSF enhancement on pcT2wFLAIR images to provide a comprehensive overview of prevalence of this new biomarker in healthy and diseased populations as well as its etiology and optimal detection methodology. We extracted information on the prevalence of CSF enhancement, its vascular risk factor and neuroimaging correlates, and methodological attributes of each study. Forty-four eligible studies were identified. By pooling data, we found that the prevalence of CSF enhancement was 82% (95% confidence interval (CI) 80–89) in meningitis (4 studies, 65 patients), 73% (95%CI 62–81) in cases with (post-) acute intracerebral hemorrhage (2 studies, 77 cases), 64% (95% CI 54–73) in cases who underwent surgery for aneurysm treatment (2 studies, 99 patients), 40% (95% CI 30–51) in cases who underwent surgery for carotid artery disease treatment (3 studies, 76 patients), 27% (95% CI 25–30) in cases with acute ischemic stroke (9 studies, 1148 patients), 21% (95% CI 17–23) in multiple sclerosis (6 studies, 897 patients), and 13% (95% CI 7–21) in adult controls (4 studies, 112 cases). Presence of CSF enhancement was associated with higher age in eleven studies, with lobar cerebral microbleeds in one study, and with cerebral atrophy in four studies. PcT2wFLAIR imaging represents a promising method that can provide novel perspectives on BBB leakage into CSF compartments, with the potential to reveal important new insights into the pathophysiological mechanisms of varying neurological diseases.

## Highlights

• CSF enhancement on post-contrast FLAIR images is a novel marker for BBB leakage.
• This neuroradiological marker is frequently observed in neurological diseases.
• Post-contrast FLAIR CSF enhancement is associated with higher age and brain atrophy.
• There is large methodological heterogeneity between studies that use this technique.
• We provide recommendations for future methodological standardization.

## 1 Introduction background

Bood-brain barrier (BBB) disruption plays a pivotal role in the pathophysiology of many neurological diseases (Starr, 2003, Cramer et al., 2015, Villringer, 2017). Disruption of the BBB can be assessed in living individuals in the form of gadolinium-based contrast agent (GBCA) leakage with contrast-enhanced magnetic resonance imaging (MRI). A novel and relatively unknown neuroradiological marker for GBCA extravasation due to presumed BBB (or blood-cerebrospinal fluid barrier) disruption is cerebrospinal fluid (CSF) enhancement on post-contrast (pc) T2-weighted fluid-attenuated inversion recovery (T2wFLAIR) images (box 1). The FLAIR signal hyperintensity following contrast administration becomes visible because the CSF is no longer fully suppressed by the inversion pulse due to local shortening of the T1-relaxation time caused by GBCA leakage into the CSF. PcT2wFLAIR has several important differences compared to pcT1-weighted (T1w) MRI, which currently is the most widely-used and conventional method for the assessment of BBB leakage (box 2) (Raja et al., 2018, Thrippleton, 2019). Because of its unique properties, pcT2wFLAIR imaging represents a promising method to gain novel insights into the dynamics and extent of BBB leakage and CSF-clearance mechanisms in aging and neurological diseases.

Box 1 **: Definitions of terminology used throughout the paper**

**Blood-brain barrier (BBB) disruption**: Extravasation of blood constituents (including gadolinium-based contrast agents (GBCAs)) into the brain tissue and surrouning fluid-filled spaces.

**CSF enhancement**: The radiologic finding of hyperintense signal within the cerebrospinal and/or interstitial fluid space, including the ventricles, subarachnoid space and perivascular space, on post-contrast T2-weighted FLAIR images, presumably caused by GBCA extravasation due to BBB disruption.

Although first published reports of pcT2wFLAIR CSF enhancement date back to the late 90′s (Lev and Schaefer, 1999, Mathews, 1999), only few studies have been published on this topic ever since. In contrast, since the first GBCAs became available for clinical use globally during the late 80′s (Lohrke, 2016), a large number of studies were published on BBB leakage measured with pcT1-weighted imaging. A potential reason for this difference in popularity may simply be that most studies focus on the brain parenchyma, in which pcT1-weighted imaging is known to be more sensitive to detect GBCA leakage than pcT2wFLAIR. However, the number of studies using pcT2wFLAIR imaging has substantially increased in recent years, providing a new perspective on BBB leakage in healthy and diseased populations.

**Fig. 1:**
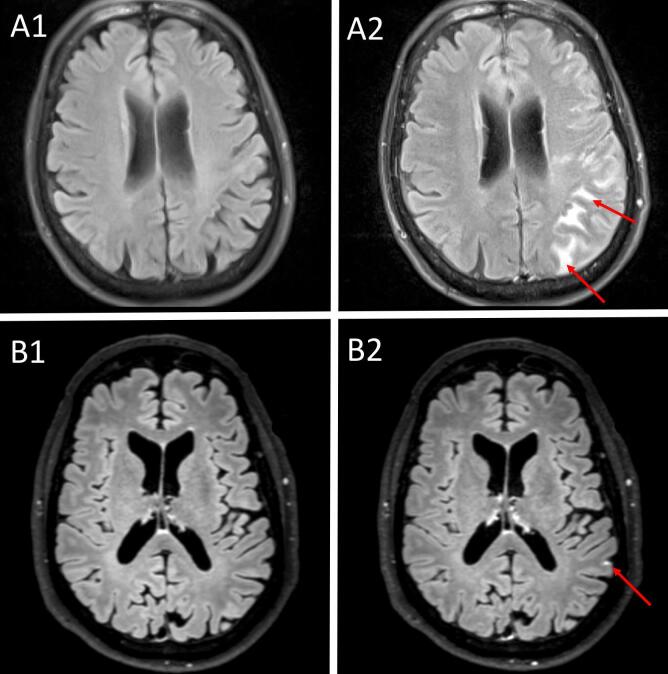
Different appearances of post-contrast T2-weighted FLAIR cerebrospinal fluid enhancement. The top row shows pre- (A1) and post- (A2) contrast T2-weighted FLAIR images in a stroke patient. The post-contrast image was acquired approximately 24 h after contrast administration and within 48 h after stroke onset, and shows widespread sulcal hyperintensities [Reprinted with permission from Springer Nature, European Radiology; license number 4818791304726 (Ostwaldt, 2015). The bottom row shows pre- (B1) and post- (B2) contrast T2-weighted FLAIR images in a cognitively normal older individual (Freeze, 2017). The post-contrast image was acquired approximately 16 min after contrast administration and shows a focal punctate hyperintensity. All images were acquired at a field strangth of 3 T. Note that the difference in appearance can possibly be attributed to individual case characteristics, but also to the difference in post-contrast acquisition timing.

Previous studies have applied pcT2wFLAIR to demonstrate GBCA leakage in the form of CSF enhancement in various neurological diseases with presumed BBB disruption, but also in non-diseased controls (Freeze, 2017, Absinta, 2017). The appearance of these MRI signal abnormalities is highly variable and can range from very subtle single-focus or multi-focal pericortical signal enhancement in memory clinic patients, to more widespread enhancement in patients with acute ischemic stroke (Fig. 1). It is unclear how frequent this neuroradiological phenomenon appears in different disease populations and there is currently no consensus regarding its terminology, definition and optimal detection methodology. Moreover, it is unknown whether pcT2wFLAIR CSF enhancement is associated with common vascular risk factors, neuroradiological imaging markers of vascular and neurodegenerative disease, or a poor prognosis. In this systematic review we aimed to provide a comprehensive overview of published studies on pcT2wFLAIR CSF enhancement.

Box 2 **: Important differences between pcT2wFLAIR and pcT1w imaging.**

• pcT2wFLAIR imaging is more suitable for the detection of subtle GBCA leakage into the CSF compared to pcT1w imaging, with up to 10 times higher sensitivity at low CSF gadolinium concentrations (Freeze, 2017, Kohrmann et al., 2012).
• in contrast to pcT1wMRI, pcT2wFLAIR imaging is relatively insensitive to GBCA leakage into the brain parenchyma, which can be attributed to its T2-weighted component (Freeze, 2017, Mamourian et al., 2000).
• pcT2wFLAIR is insensitive to high GBCA concentrations in blood vessels, and as such allows for the ‘pure’ detection of BBB leakage of GBCA in the CSF without interference from high signal due to high GBCA concentrations in leptomeningeal blood vessels that is typically observed on pcT1w imaging.

## 2 Methods

### 2.1 Literature search strategy

We identified studies through a systematic search of MEDLINE (1946-January 29, 2020) and EMBASE (1974-January 29, 2020) (appendix e-1). We followed the PRISMA guideline (Preferred Reporting Items for Systematic Reviews and Meta-Analyses).

### 2.2 Inclusion and exclusion criteria

We included human *in vivo* MRI-studies of the brain in which a T2wFLAIR sequence was performed after administration of a GBCA with visual assessment of leakage (signal enhancement) within the CSF space (including the subarachnoid space, ventricles, and perivascular spaces). We excluded conference reports, comments, case reports (defined as n < 5 cases), reviews, and articles in languages other than English. We excluded studies that assessed enhancement on T1wFLAIR images, studies that applied contrast administration intrathecally, studies that only included participants < 18 years old, studies that assessed ocular enhancement, and studies that examined enhancement surrounding circumventricular organs (structures lining the cavity of the third and fourth ventricle, which facilitate communication between the central nervous system and peripheral blood; these structures do not have a BBB). In addition, we excluded studies in cases with tumors because the blood-tumor barrier is known to have distinct features compared to the BBB (including increased permeability) (Arvanitis et al., 2020). If a study included both cases with tumors as well as cases eligible for inclusion in our review, we included the study if extraction of data was possible for eligible cases separately.

### 2.3 Selection of studies

Title and abstract screening to select studies that were potentially eligible for inclusion was performed in duplicate by two independent authors (W.M.F. and M.vd.T.), who subsequently reviewed full-text versions of these studies. One case of uncertainty with regard to abstract and full-text screening was discussed with a third author (S.J.v.V.). The reference lists of the included articles were screened for additional papers. Two studies reported findings from exactly the same ischemic stroke case series (duplicate dataset), and therefore we pooled the results of these studies for the purpose of this review (Warach and Latour, 2004, Latour et al., 2004).

### 2.4 Study quality assessment

We assessed the methodological quality of the studies based on a version of the Newcastle Ottawa Scale that was adapted for case series studies (Herzog, 2013, Wells, 2017). In addition, we critically appraised the literature by discussing potential bias in the individual studies.

### 2.5 Data extraction

Data were extracted from the included studies using a prespecified and piloted data file by two independent authors (W.M.F. and M.vd.T). Discrepancies were resolved in consensus between these authors. The primary outcome variable of interest was the prevalence of pcT2wFLAIR CSF enhancement in each study sample. In addition, we extracted the following data: (1) details on the study sample (disease type, mean/median age, % female) and study design (cross-sectional/longitudinal and retrospective/prospective), (2) definition/terminology and location/appearance of pcT2wFLAIR CSF enhancement, (3) MRI acquisition details, (4) reports of associations with possible (vascular) risk factors (including age, sex, hypertension, diabetes mellitus, hyperlipidemia, and smoking) and conventional vascular and neurodegenerative neuroimaging markers (including white matter hyperintensities (WMH), lacunar infarcts, cerebral microbleeds and cerebral atrophy), and (5) assessment of a clinical outcome variable at least 1 month after presentation with pcT2wFLAIR CSF enhancement (to assess the prognostic value of CSF enhancement).

### 2.6 Data analysis

Because prevalence of pcT2wFLAIR CSF enhancement was the primary outcome variable of interest, we computed pooled prevalence estimates for broadly comparable disease groups when possible. When computing the pooled prevalence estimates, we only included individual studies that did not select their study sample based on CSF enhancement positivity (i.e., non-selective studies). When there was known or suspected overlap between study samples, we included the study with the largest study sample in the pooled data presentation (i.e., non-overlapping studies). We calculated the prevalence of CSF enhancement by dividing the number of cases with CSF enhancement by the total number of study participants. We obtained 95% confidence intervals (95% CI) using the modified Wald method with the DescTool package in R statistical software (R version 3.6.1) (Agresti and Coull, 1998, R Core Team. R: A, 2016). In addition, we computed the pooled mean age for each disease group by taking the sum of the mean age (if not available, the median was taken instead) * number of participants of each study divided by the total number of study participants.

## 3 Results

**Fig. 2:**
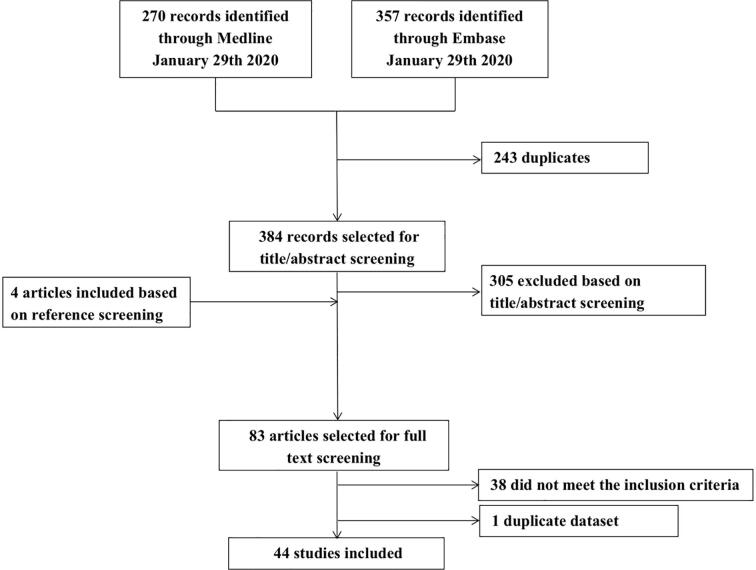
Flow chart of study selection.

Forty-four out of 388 unique publications met the inclusion criteria (Fig. 2). Most studies were performed in cases with ischemic stroke, transient ischemic attack (TIA), and/or intracerebral hemorrhage (ICH) (n = 20) (Villringer, 2017, Ostwaldt, 2015, Ostwaldt, 2014, Warach and Latour, 2004, Latour et al., 2004, Choi, 2017, Dechambre, 2000, Forster et al., 2016, Gupta, 2017, Henning et al., 2008, Hjort, 2008, Kim, 2005, Luby, 2019, Nadareishvili, 2018, Lee, 2015, Lee, 2016, Lee, 2018, Rozanski, 2010, Kidwell et al., 2011, Jolink, et al., 2019, Barr, 2010). Six studies described patients who underwent cardiovascular or intracranial vascular surgery (Cho, 2014, Ogami et al., 2011, Wilkinson, 2000, Li, 2018, Suthiphosuwan et al., 2018, Merino, 2013), nine studies patients with multiple sclerosis (MS) (one of these included stroke cases as a positive control (Eisele, 2015) (Eisele, 2015, Absinta, 2015, Bergsland, 2019, Harrison, 2017, Jonas et al., 2018, Zivadinov, 2017, Zivadinov, 2018, Zurawski, 2020, Ighani, 2020), four studies patients with meningitis (Ahmad et al., 2015, Alonso, 2015, Fukuoka, 2010, Splendiani, 2005), two studies reported on memory clinic patients and normal aging (Freeze, 2017, Freeze et al., 2019), one study on Susac syndrome and MS (Coulette, 2019), one study on familial amyloid polyneuropathy (Hirai, 2005), and one study included a mix of diseases, which were presented as follows: inflammatory and immune-mediated neurologic diseases (neuromyelitis optica spectrum disorder, immune-mediated encephalitis, immune-mediated cerebellar ataxia, systemic inflammatory diseases with white matter MRI abnormalities not suggestive for MS and Susac syndrome), noninflammatory neurologic diseases (small vessel disease, migraine, neurodegenerative diseases, compressive myelopathy), human T-lymphotropic virus (HTLV) infection, and human immunodeficiency virus (HIV) infection (Absinta, 2017).

### 3.1 Methodological quality of the individual studies

The methodological quality of the studies as assessed with the adapted Newcastle-Ottawa scale (Herzog, 2013, Wells, 2017) varied from poor (score 3/8) to excellent (score 8/8) (details of the study quality assessment can be found in supplemental table e-1). The average quality score for studies in patients with stroke, post-cardiovascular and intracranial vascular surgery, and meningitis was moderate (score 5/8), while studies in patients with MS had a higher average quality score (score 7/8).

Bias, confounding factors and chance should be considered when interpreting the results of the individual studies. Inherent to the nature of the method, all studies of pcT2wFLAIR enhancement were subject to selection bias because participants needed to be eligible for MRI and GBCA administration (patients that are typically excluded are those with cardiac pacemakers and renal failure). Apart from this, 23 studies included cases that were reasonably representative of their population (i.e. (semi-) consecutive case series or prospective cohort study), but 21 studies used mostly retrospectively collected data with insufficient details on case selection procedures, and three of these selected only cases with CSF enhancement positivity (supplemental table e-1 and e-2). In addition, some studies did not mention that the assessment of CSF enhancement was performed while blinded to clinical data (20 studies). Confounding factors may have influenced the results within several studies, including failure to acquire pre-contrast FLAIR scans for all participants (15 studies), variable post-contrast image acquisition delay times (14 studies), and variable contrast agent dosage within the studied samples (11 studies) (supplemental table e-1 and e-5). Finally, results of studies with smaller sample sizes are more susceptible to chance (13 studies included n < 30).

### 3.2 Prevalence of CSF enhancement

#### 3.2.1 Stroke

**Fig. 3:**
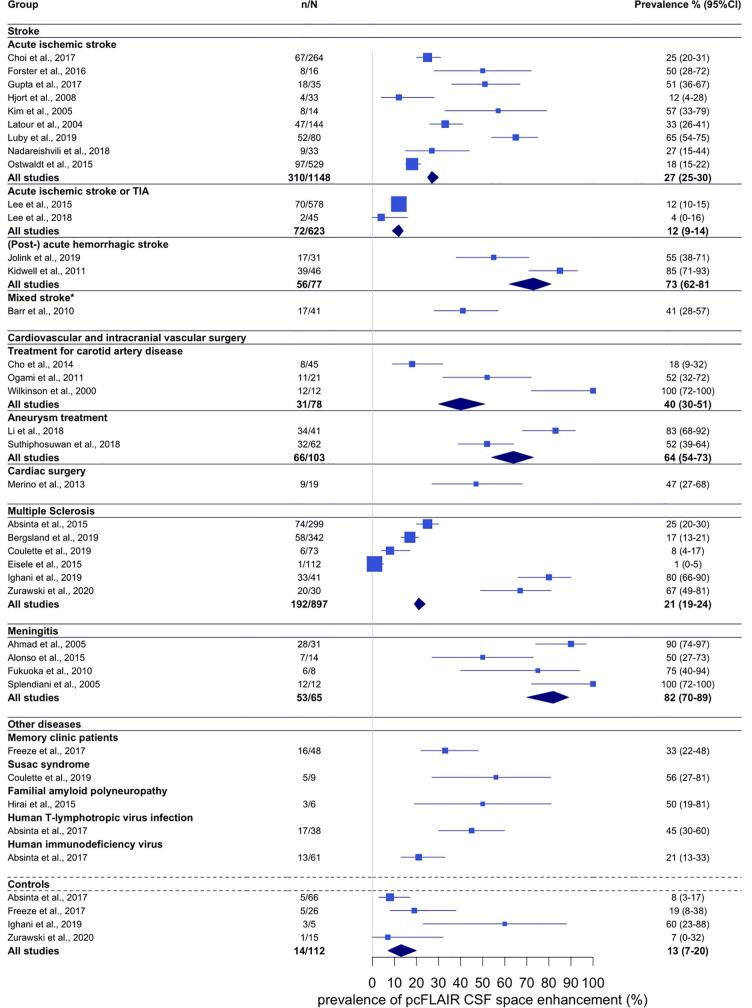
The prevalence of pcT2wFLAIR CSF enhancement across disease groups. Point estimates are represented by squares for individual studies, and diamonds for summed disease categories. The area of each point estimate is proportional to the size of each study. The error bars represent 95% confidence intervals. N = sample size, n = number of cases with pcT2wFLAIR CSF enhancement. * Study sample included both cases with ischemic stroke as well as cases with intracerebral hemorrhage.

Twenty-one studies assessed CSF enhancement in cases with stroke (supplemental table e-2). Fourteen studies examined acute ischemic stroke, including nine studies with non-selective and non-overlapping study samples with a total of 1148 cases and a pooled mean age of 70 years (Ostwaldt, 2015, Warach and Latour, 2004, Latour et al., 2004, Choi, 2017, Forster et al., 2016, Gupta, 2017, Hjort, 2008, Kim, 2005, Luby, 2019, Nadareishvili, 2018). The reported prevalence of CSF enhancement in these studies ranged from 12% to 65%, with a pooled prevalence estimate of 27% (95% CI 25–30). Four studies included a mix of cases with acute ischemic stroke and TIA, including two studies with non-overlapping study samples in 623 cases and a pooled mean age of 60 years (Lee, 2015, Lee, 2018). These studies reported prevalence estimates of 12% and 4%, corresponding to a pooled prevalence estimate of 12% (95% CI 9–14) when combined. Two studies in a total of 77 cases with lobar or deep ICH and a pooled mean age of 63 years reported higher prevalence numbers of 54% (post-acute ICH) (Jolink, et al., 2019)and 85% (acute ICH) (Kidwell et al., 2011), with a pooled prevalence estimate of 73% (95% CI 62–81). One additional study included a mix of 41 cases (mean age 62 years) with acute ischemic stroke, TIA, and ICH (Barr, 2010), and reported a prevalence of 41% (Fig. 3).

#### 3.2.2 Cardiovascular or intracranial surgery

Six studies assessed CSF enhancement in the acute phase after cardiovascular or intracranial vascular surgery (supplemental table e-2). Among these were three studies in a total of 76 cases with a pooled mean age of 70 years who underwent carotid stenting or carotid endarterectomy in the context of carotid artery disease, which reported prevalence numbers of 18% (Cho, 2014); 54% (Ogami et al., 2011); and 100% (Wilkinson, 2000). When these studies were combined, the pooled prevalence estimate was 40% (95% CI 30–51). Two studies examined a total of 99 cases with a pooled mean age of 58 years who underwent unruptured aneurysm treatment and reported prevalence numbers of 52% (Suthiphosuwan et al., 2018)and 83% (Li, 2018), with a combined prevalence estimate of 64% (95% CI 54–73). In addition, one study in 19 cases (mean age 67 years) who underwent cardiac surgery reported a prevalence of 47% (Merino, 2013)(Fig. 3).

#### 3.2.3 Multiple sclerosis

Nine studies assessed CSF enhancement in MS, of which six with non-overlapping study samples including 897 cases with a pooled mean age of 44 years (supplemental table e-2) (Zurawski, 2020, Ighani, 2020, Coulette, 2019, Eisele, 2015, Absinta, 2015, Bergsland, 2019). The reported prevalence estimates within these studies ranged between 1% and 80%, with a pooled prevalence estimate of 21% (95% CI 19–24). Because of the wide variation in reported prevalence we inspected the extracted data and found that the wide variation seemed to be related to magnetic field strength differences between studies. Studies that used 3 T MRI reported prevalence estimates between 1% and 25% (Eisele, 2015, Bergsland, 2019, Coulette, 2019), while studies that used 7 T MRI report estimates of 67% (Zurawski, 2020)and 80% (Ighani, 2020).

#### 3.2.4 Meningitis

Four studies in 65 cases with meningitis with mixed etiological causes (viral, bacterial, fungal) with a pooled mean age of 47 years reported prevalence estimates between 50% and 100%, resulting in a pooled prevalence estimate of 82% (95% CI 70–89) (supplemental table e-2) (Ahmad et al., 2015, Alonso, 2015, Fukuoka, 2010, Splendiani, 2005).

#### 3.2.5 Other diseases

For several other diseases it was not possible to assess pooled prevalence estimates because only single or overlapping studies were found. Among these was one study in 48 memory clinic patients (mean age 70 years) reporting a prevalence estimate of 33% (Freeze, 2017). One study in nine cases with Susac syndrome (mean age 38 years) reported a prevalence estimate of 56% (Coulette, 2019). Another study in familial amyloid polyneuropathy (mean age 40 years) found CSF enhancement in three out of six cases (50%) (Hirai, 2005). In addition, we included one study that reported a prevalence of 35% in a mixed group of 51 cases (mean age 43 years) with diagnoses of neuromyelitis optica spectrum disorder, immune-mediated encephalitis, immune-mediated cerebellar ataxia, systemic inflammatory diseases with white matter MRI abnormalities not suggestive for MS, and Susac syndrome, a prevalence of 8% in a group of 38 cases (mean age 50 years) with mixed noninflammatory neurologic diseases, including small vessel disease, migraine, neurodegenerative diseases, and compressive myelopathy, a prevalence of 45% in 38 cases with HTLV infection (mean age 53 years), and a prevalence of 21% among 61 cases with HIV infection (mean age 52 years) (supplemental table e-2) (Absinta, 2017).

#### 3.2.6 Control cases

Among seven studies that included control cases, four had non-overlapping study samples including a total of 112 cases (mean age 50 years, supplemental table e-3) (Freeze, 2017, Absinta, 2017, Zurawski, 2020, Ighani, 2020). The reported prevalence numbers of CSF enhancement ranged between 7% and 60%, although the latter study included only five participants. The pooled prevalence estimate of the controls in all studies combined was 13% (95% CI 7–20) (Fig. 3).

### 3.3 Terminology of CSF enhancement

Most studies referred to pcT2wFLAIR CSF enhancement with either the term ‘HARM’ (hyperintense acute reperfusion marker) or the term ‘LME’ (leptomeningeal enhancement) (supplemental table e-4). The term HARM was introduced in 2004 by Warach and Latour because they hypothesized that enhancement of the CSF space in acute ischemic stroke was related to reperfusion injury (Warach and Latour, 2004). Since then, many studies in acute ischemic stroke (Villringer, 2017, Ostwaldt, 2015, Ostwaldt, 2014, Choi, 2017, Forster et al., 2016, Gupta, 2017, Henning et al., 2008, Hjort, 2008, Luby, 2019, Nadareishvili, 2018, Lee, 2015, Lee, 2016, Lee, 2018, Rozanski, 2010), but also studies in hemorrhagic stroke (Kidwell et al., 2011, Jolink, et al., 2019), treatment for carotid artery disease (Cho, 2014), and cardiac surgery (Merino, 2013), have adopted the term. By comparison, the term ‘LME’ refers to the abnormal accumulation of contrast media in the pia mater, arachnoid mater, and/or the subarachnoid space, and has also been used by studies in a variety of diseases, including ischemic stroke (Wilkinson, 2000), MS (Absinta, 2015, Harrison, 2017, Coulette, 2019, Coulette, 2019, Zurawski, 2020, Ighani, 2020, Ahmad et al., 2015, Alonso, 2015, Fukuoka, 2010, Splendiani, 2005, Freeze et al., 2019), and meningitis (Ahmad et al., 2015, Alonso, 2015, Fukuoka, 2010).

### 3.4 Definition of CSF enhancement

All studies that mentioned a definition of CSF enhancement acknowledged the appearance of contrast enhancement within the CSF (ventricles and/or sulci and/or cisterns) and/or leptomeninges (subarachnoid space and/or leptomeningeal compartment) (supplemental table e-4). Some studies included also parenchymal hyperintensities in their definition (and these were also reported) (Gupta, 2017, Lee, 2016, Jolink, et al., 2019, Suthiphosuwan et al., 2018). Several studies defined that the CSF enhancement needed to be brighter than cortical tissue or a superficial cortical vein with visual assessment (Freeze, 2017, Freeze et al., 2019, Absinta, 2017, Absinta, 2015, Dechambre, 2000, Suthiphosuwan et al., 2018, Bergsland, 2019, Zivadinov, 2017, Zivadinov, 2018, Coulette, 2019). Some studies required the CSF enhancement to be visible on multiple consecutive slices (Ostwaldt, 2015, Nadareishvili, 2018, Barr, 2010, Ahmad et al., 2015).

### 3.5 Appearance and location of CSF enhancement

**Fig. 4:**
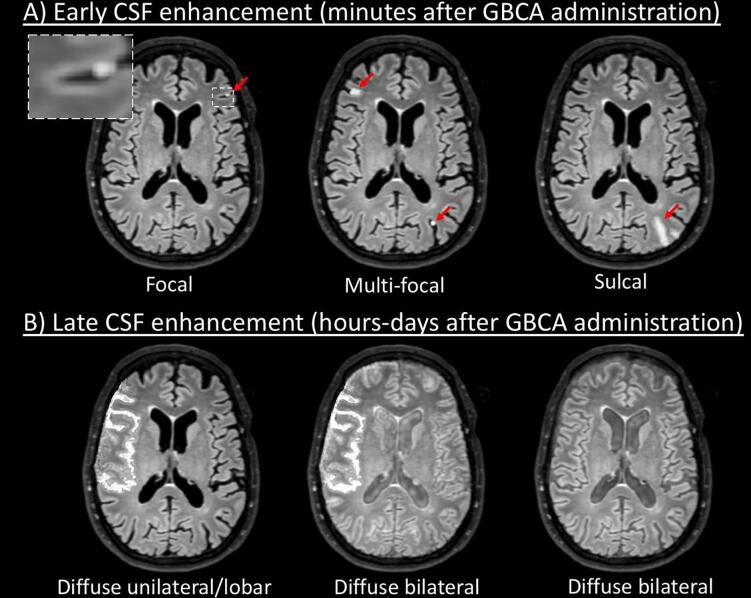
Schematic representation of different appearance patterns of CSF enhancement. A) Typical presentations of CSF enhancement within minutes after GBCA administration. These patterns were described in different diseases, including ischemic stroke, hemorrhagic stroke, carotid artery disease treatment, aneurysm treatment, cardiac surgery, multiple sclerosis, memory clinic patients, Susac syndrome (lesions were frequently observed in the posterior fossa). These types of CSF enhancement typically appear within minutes after GBCA administration. Meningitis was an exception to this: cases with meningitis typically display diffuse meningeal enhancement within minutes after GBCA administration on pcT2wFLAIR images. B) Typical presentations of CSF enhancement within hours to days after GBCA administration. Patterns like these were described in acute ischemic stroke, hemorrhagic stroke, treatment for carotid artery disease, aneurysm treatment, cardiac surgery, and familial amyloid polyneuropathy. Note: the CSF enhancement patterns were manually drawn on an example pre-contrast FLAIR image from our previous study (Villringer, 2017)using online photo editor software (www.pixlr.com).’

Manually drawn illustrative examples of the different appearance patterns of CSF enhancement are displayed in Fig. 4.

#### 3.5.1 Stroke

Descriptions of the appearance of CSF enhancement in stroke studies ranged from focal to sulcal to diffuse (supplemental table e-4). Interestingly, some studies that examined CSF enhancement at multiple timepoints showed that enhancement was absent or focal within minutes after contrast administration, while diffuse enhancement was observed after multiple hours and up to five days after initial contrast administration (Warach and Latour, 2004, Latour et al., 2004, Lee, 2015, Lee, 2016). Some studies reported a positive association between contrast agent dosage and CSF enhancement (Ostwaldt, 2015, Ostwaldt, 2014). Locations of CSF enhancement included sulci, subarachnoid space, ventricles, and cisterns. From studies in acute ischemic stroke it could be inferred that the enhancement was frequently observed in, but not necessarily restricted to, the same vascular territory and hemisphere as the infarction (Ostwaldt, 2015, Warach and Latour, 2004, Latour et al., 2004, Henning et al., 2008, Kim, 2005, Luby, 2019, Lee, 2016, Choi, 2017, Dechambre, 2000, Forster et al., 2016). In contrast, two studies in ICH reported that CSF enhancement was noncontiguous with the hemorrhage (Kidwell et al., 2011, Jolink, et al., 2019).

#### 3.5.2 Cardiovascular or intracranial surgery

CSF enhancement was described as focal, sulcal, lobar or diffuse (supplemental table e-4) (Wilkinson, 2000, Li, 2018, Merino, 2013). PcT2wFLAIR scans were acquired shortly after contrast administration or after several hours, but none of the studies assessed the course of CSF enhancement over time. Two studies in patients that underwent treatment for carotid artery disease described the enhancement as being primarily located within the territory of the middle cerebral artery (Ogami et al., 2011, Wilkinson, 2000).

#### 3.5.3 Multiple sclerosis

CSF enhancement appeared as single or multiple foci in MS (supplemental table e-4). All studies assessed CSF enhancement shortly (i.e., within 10–20 min) after contrast administration. Three studies used a subtraction method of pre- and post-contrast FLAIR scans to detect CSF enhancement (Harrison, 2017, Zivadinov, 2018, Ighani, 2020). The majority of foci were located supratentorially (Ighani, 2020, Coulette, 2019, Jonas et al., 2018, Zivadinov, 2017, Zivadinov, 2018). Two studies performed longitudinal imaging and found that the majority of foci (>82%) appeared stable over a time period of multiple years (Absinta, 2015, Jonas et al., 2018).

#### 3.5.4 Meningitis

CSF enhancement in cases with meningitis was described as circumscribed or diffuse, either within the sulci along each cerebral lobe of both hemispheres and the cerebellum, or confined to the supratentorial leptomeninges (supplemental table e-4) (Alonso, 2015, Fukuoka, 2010, Splendiani, 2005). All studies assessed CSF enhancement within minutes after contrast administration.

#### 3.5.5 Control cases

CSF enhancement in control cases was assessed within minutes after contrast administration and described as focal (supplemental table e-4) (Freeze, 2017, Freeze et al., 2019, Absinta, 2017, Absinta, 2015, Zurawski, 2020, Ighani, 2020). On follow-up imaging after >1 year the foci remained stable (Freeze, 2017, Freeze et al., 2019).

### 3.6 MRI details

The MRI acquisition details, contrast dosage, and contrast agent type were highly variable between studies (supplemental table e-5). The magnetic field strength ranged from 1.5 T (T) (n = 14 studies), a mix of 1.5 T and 3 T (n = 6 studies), 3 T (n = 18 studies), to 7 T (n = 5 studies), and one study did not mention field strength (Lee, 2018). The FLAIR sequence parameters varied significantly, even between studies using the same magnetic field strength, which can possibly be attributed to manufacturer or site preferences. For example, the echo time of studies performed at 3 T ranged between 100 and 500 ms. Slice thickness ranged between 0.7 and 7.0 mm, where specified; 11 studies did not mention slice thickness. Contrast agent types that were used included gadobutrol (10 studies), gadopentetate (12 studies), gadoterate (4 studies), gadobenate (2 studies), gadoteridol (3 studies), gadodiamide (1 study), some studies used two different types of contrast agents on different cases (n = 2 studies), and 10 studies did not mention the contrast agent type. Contrast agent doses that were applied also varied, although most studies used a single dose (0.1 mmol/kg, n = 26 studies). One study applied a double dose (0.2 mmol/kg), two studies used a double dose or a fixed dose of 15 ml gadobutrol (which translates to a double dose for a 75 kg person; vial concentration, 1 mmol/ml), several studies reported a fixed dose (15 ml gadopentetate (0.5 mmol/ml); 20 ml gadobenate (0.5 mmol/ml); 4–6 ml or 10 ml gadobutrol), six studies used variable or multiple contrast agent administrations, and five studies did not mention the dosage. Because of the large heterogeneity in study samples/diseases and MRI acquisition details we did not set out to explore associations between specific imaging parameters and prevalence of CSF enhancement.

### 3.7 Risk factor and neuroradiological correlates

Eleven out of 21 studies found that CSF enhancement increased with increasing age (supplemental table e-6) (Freeze, 2017, Ostwaldt, 2015, Warach and Latour, 2004, Latour et al., 2004, Lee, 2015, Rozanski, 2010, Barr, 2010, Ogami et al., 2011, Absinta, 2015, Zivadinov, 2018). Studies that assessed sex (n = 19), hypertension (n = 12), diabetes mellitus (n = 10), hyperlipidemia (n = 6) or smoking (n = 6) as potential risk factors for CSF enhancement did not find any significant associations, except for two studies that found a negative association between CSF enhancement and hyperlipidemia (Rozanski, 2010)or diabetes mellitus (Gupta, 2017). CSF enhancement was not associated with WMH in six studies (Freeze, 2017, Rozanski, 2010, Jolink, et al., 2019, Cho, 2014, Absinta, 2015, Ighani, 2020), but one study in MS reported a higher volume of hyperintense lesions in the white matter in cases with CSF enhancement compared to cases without CSF enhancement (Zurawski, 2020). One study that assessed the relationship between CSF enhancement and lacunar infarcts did not find a significant association (Freeze, 2017). Of the two studies that assessed the association between CSF enhancement and microbleeds, one study did not find an association (Freeze, 2017), while another study in cases with ICH reported that cases with CSF enhancement more often had lobar cerebral microbleeds (13/17, 77%) than those without (5/14, 36%) (Jolink, et al., 2019). In contrast, no association was found between CSF enhancement and non-lobar microbleeds (Jolink, et al., 2019). Of seven studies that assessed associations between CSF enhancement and atrophy, three found no association (Freeze, 2017, Absinta, 2017, Zurawski, 2020), while four MS studies found positive associations between atrophy and CSF enhancement (Absinta, 2015, Bergsland, 2019, Zivadinov, 2017, Ighani, 2020).

### 3.8 Prognostic value

Two studies in ischemic stroke found that CSF enhancement was associated with worse clinical outcome 1–3 months after the event as indicated by higher modified Rankin Scales (mRS) in patients with compared to those without CSF enhancement (Ostwaldt, 2015, Warach and Latour, 2004, Latour et al., 2004). One study of patients with TIA found that after 90 days the median mRS was 0 for both cases with and without CSF enhancement (Lee, 2015). No information was found on the prognostic value of CSF enhancement in other diseases.

## 4 Discussion

CSF enhancement on pcT2wFLAIR images is a frequent finding in multiple conditions and diseases, including post-vascular surgery conditions, stroke, and MS, though CSF enhancement can also be found in non-neurological controls. Increasing age was identified as a possible risk factor for CSF enhancement in multiple studies, while other classical vascular risk factors were not. CSF enhancement is associated with cerebral atrophy in patients with MS, and may be associated with cerebral amyloid angiopathy in patients with ICH. The clinical and prognostic value of CSF enhancement remains unclear.

We encountered risk of bias within the individual studies, including selection bias, presence of confounding factors, and limited power. Other factors that might have impacted the frequency estimates included failure to acquire pre-contrast FLAIR images in all participants and potential unawareness of neuroradiological mimics of GBCA enhancement in the CSF space (Stuckey et al., 2007), including enhancement in or near dural venous sinuses (Absinta, 2017, Absinta, 2015, Harrison, 2017, Absinta, et al., 2017), basal meninges or large subarachnoid veins (Absinta, 2017, Absinta, 2015, Harrison, 2017), enhancement due to (subarachnoid) hemorrhage (Ostwaldt, 2015, Lee, 2015), or hyperoxygenation (Suthiphosuwan et al., 2018). We have attempted to minimize the limitations of the individual studies by pooling the results of broadly comparable disease groups. At the cross-study level, we observed large variations with regard to study samples, MRI sequence parameters, contrast agent dosage, and time delay between contrast agent administration and pcT2wFLAIR acquisition. Moreover, many studies failed to report one or more of these important methodological aspects in detail. The methodological variability between studies is reflected by the large heterogeneity in prevalence estimates among studies with broadly comparable disease groups. This, together with the absence of a common terminology and neuroradiological definition of CSF enhancement, makes it challenging to directly compare studies. In the literature we found two frequently used terms for CSF enhancement, including ‘hyperintense acute reperfusion marker’ (HARM) and ‘leptomeningeal enhancement’ (LME). However, these terms may not be universally applicable since it remains unclear whether reperfusion constitutes the main mechanism underlying CSF enhancement, and enhancement has also been observed in CSF spaces beyond the subarachnoid space. Therefore, we propose using the neutral term ‘cerebrospinal fluid (CSF) enhancement’.

The optimal imaging acquisition methodology to capture CSF enhancement remains unclear. Because of the large heterogeneity in imaging methodology and study samples we did not formally assess associations between the prevalence of CSF enhancement and specific imaging aspects, though from our experience and extensive review of the literature we infer several factors that are likely to influence the conspicuity of low GBCA concentrations in the CSF. Firstly, several studies have demonstrated that the likelihood and conspicuity of CSF enhancement on pcT2wFLAIR images increases with higher contrast agent dosage or with multiple doses (Mamourian et al., 2000, Ostwaldt, 2015, Ostwaldt, 2014, Kidwell et al., 2011, Wilkinson, 2000). On a related note, the rate of GBCA excretion, which is dependent on renal functioning, might also affect the appearance of CSF enhancement, especially in studies with long post-contrast acquisition delays. Secondly, we recently demonstrated that the conspicuity of CSF enhancement due to low GBCA concentrations is strongly dependent on the parameters of the pcT2wFLAIR sequence, and especially benefits from a longer echo time (Freeze, 2019). Thirdly, 2D pcT2wFLAIR sequences are likely insensitive for the detection of subtle focal CSF enhancement due to the thicker slices compared with 3D sequences with isotropic voxel sizes (Eisele, 2015). Fourthly, MS studies performed at 7 T found higher frequencies of CSF enhancement compared to studies performed at 3 T (Eisele, 2015, Absinta, 2015, Coulette, 2019, Harrison, 2017, Jonas et al., 2018, Zivadinov, 2017, Zivadinov, 2018, Zurawski, 2020, Ighani, 2020), which suggests that the magnetic field strength influences the conspicuity of CSF enhancement (possibly due to an increased signal-to-noise ratio). Fifthly, one study has recently demonstrated the added value of using pre- and post-contrast subtraction images for the detection of focal pericortical enhancement, as this method avoided false-positives and increased the accuracy detecting focal CSF enhancement (Zivadinov, 2018). Finally, it currently remains unclear what the optimal acquisition timing for the detection of CSF enhancement is in different diseases. It is important to be aware that post-contrast acquisition timing likely influences the CSF enhancement appearance, as stroke studies with multiple acquisition moments showed that absent or focal enhancement can transform in widespread diffuse signal enhancement throughout the subarachnoid space at later timepoints (Warach and Latour, 2004, Latour et al., 2004, Lee, 2015, Lee, 2016). Many questions remain as to how CSF enhancement appears on differently timed pcFLAIR acquisitions. For example, it remains unknown whether focal leakage observed within minutes after GBCA administration can transform into diffuse CSF enhancement at later timepoints in diseases other than stroke. In addition, while studies in MS and memory clinic patients show that CSF enhancement can be a chronic phenomenon in these diseases, no studies to date have performed follow-up imaging in the chronic phase of ischemic stroke. Moreover, it remains unclear whether and how the appearance of CSF enhancement in acute stroke (and perhaps also post-surgical conditions) is associated with the elapsed time between the event and imaging, as post-stroke BBB opening is considered to be a dynamic process (Sandoval and Witt, 2008).

Interestingly, the finding of widespread contrast agent retention in the subarachnoid space up to several days after contrast administration in multiple studies of stroke cases suggests that CSF enhancement forms a potential biomarker for impaired CSF dynamics and/or clearance deficiencies (glymphatics) (Deike-Hofmann, 2019, Lee, 2018), as the appearance of CSF enhancement is influenced by a mix of BBB opening and CSF washout. The increased sensitivity of pcT2w FLAIR to low GBCA concentrations compared with pcT1w imaging, as demonstrated by previous studies (Mathews, 1999, Freeze, 2017, Kohrmann et al., 2012), makes this technique especially suitable for tracking the circulation of GBCA through the various CSF compartments. Previous work has demonstrated the feasibility of tracking changes in CSF signal intensity over time in the ventricles, perivascular spaces, and cisterns, both in control participants and patients with BBB impairment due to cerebral metastases (Deike-Hofmann, 2019). Furthermore, recent studies have shown that glymphatic functioning in the form of GBCA clearance from the CSF can be investigated with repeated pcT2wFLAIR imaging after intrathecal GBCA administration (Ringstad and Eide, 2020, Ringstad et al., 2017).

Several studies reported on associations between possible (vascular) risk factors and neuroradiological features. Among the examined risk factors, higher age stood out as a universal risk factor among neurological study samples (n = 11/20 studies), which is in line with previous studies that found associations between BBB leakage and increasing age (Farrall and Wardlaw, 2009). However, none of the included studies looked at the association between CSF enhancement and age within non-neurological controls so it is possible that the observed associations can be explained by increases in disease severity and/or duration with age. Nevertheless, age should likely be considered an important confounding variable when comparing pooled prevalence estimates, especially between disease groups and controls.

None of the included studies reported significant positive associations between CSF enhancement and sex or vascular risk factors. With regard to neuroradiological correlates, one study in post-acute ICH patients found a positive association between CSF enhancement and the presence of lobar cerebral microbleeds (Jolink, et al., 2019), suggesting a role for that cerebral amyloid angiopathy in BBB disruption, in line with neuropathological observations and other previous studies (Freeze, 2019, Freeze, 2018). In addition, one study in MS reported a higher volume of white matter hyperintense lesions in cases with CSF enhancement compared to those without, and four studies in MS reported increased atrophy in cases with compared to cases without CSF enhancement. Together, these findings suggest that inflammatory and/or neurodegenerative processes might play an important role in the formation of CSF enhancement. This hypothesis is supported by one study that showed perivascular inflammation at the location where CSF enhancement was observed during life in two MS patients who came to autopsy (Absinta, 2015). We found only three studies that assessed the prognostic value of CSF enhancement over a time course of >1 month, and two of these indicated worse clinical performance in ischemic stroke cases.

Future prospective studies should further explore the clinical and prognostic significance of CSF enhancement in different disease types while including appropriate (age-matched, sufficient sample size, and scanned under the same conditions as diseased cases) control samples to determine the relative prevalence of CSF enhancement, as well as its pathological correlates. Longitudinal studies that assess CSF enhancement over a time course of several hours to days could shed light on the dynamics between BBB leakage on the one hand and CSF flow and clearance mechanisms on the other hand. The optimal methodological parameters to detect subtle GBCA leakage in CSF should be explored at different field strengths. Furthermore, future studies should explore automated methods to detect CSF enhancement on pcT2wFLAIR images for improved visibility and quantification. To facilitate increased quality and comparison between studies, we have formulated recommendations (box 3) for future studies.

Box 3 **: Recommendations for future studies.**

Suggested standardized terminology: Cerebrospinal fluid (CSF) enhancement.

Suggested standardized definition: Signal enhancement within cerebrospinal fluid compartments on T2-weighted FLAIR images after intravenous administration of a GBCA; this enhancement may be detected up to several days after GBCA administration.

Recommended standardized methodology:

• Acquire pre-contrast FLAIR images in all cases to rule out potential mimics of CSF enhancement (Absinta, 2015, Stuckey et al., 2007).
• Use a relatively long echo time (e.g. ∼ 500 ms at 3 T (Freeze et al., 2019) for increased sensitivity to lower GBCA concentrations in CSF.
• Use a 3D FLAIR sequence to increase spatial resolution and thus sensitivity to focal CSF hyperintensities (e.g. resolution ≤ 1 mm isotropic).
• Administer GBCA only once using a standard single dose and maximize safety when choosing a contrast agent type (e.g. use a macrocyclic GBCA, exclude cases with renal failure).
• Standardize timing of pcT2wFLAIR acquisition and, if possible, acquire FLAIR images at multiple time points after contrast administration (e.g. standardized after 10 min and repeatedly acquire sequence with intervals of several hours to days) to evaluate the appearance and location of CSF enhancement over time.
• Include an appropriate control sample (e.g. matched on relevant variables such as age, sufficient sample size) to determine the relative frequency of CSF enhancement.

## Financial disclosures

Whitney Freeze – Reports support from Alzheimer Nederland (grant WE.03-2018-13).

Merel van der Thiel – Reports no disclosures.

Jeroen de Bresser – Reports support from Alzheimer Nederland (grant WE.03-2019-08).

Catharina Klijn – Reports support from a clinical established investigator grant of The Netherlands Heart Foundation (grant number 2012T0770) and an ASPASIA grant from ZonMw (grant number 015008048).

Ellis van Etten – Reports no disclosures.

Jacobus Jansen – Reports no disclosures.

Louise van der Weerd – Reports no disclosures.

Heidi Jacobs – Reports no disclosures.

Walter Backes – Reports no disclosures.

Susanne van Veluw – Reports support from NWO (VENI grant 91619021).
