# Supplementary material for: CSF enhancement on post-contrast fluid-attenuated inversion recovery images; a systematic review
Source: Neuroimage Clin. 2020 Oct 2;28:102456. doi: 10.1016/j.nicl.2020.102456 (PMC7559862; doi:10.1016/j.nicl.2020.102456)
Supplement: Supplementary data 1 [file mmc1.pdf]

APPENDIX e-1

Search strategy

1. $FLAIR.ab,kw,ti.

2. $fluid attenuated inversion recovery.ab,kw,ti.

3. 1 or 2

4. (contrast adj10 agent).ab,kw,ti.

5. post?contrast.ab,kw,ti.

6. post?gadolinium.ab,kw,ti.

7. contrast?enhanc$.ab,kw,ti.

8. gadolinium$.ab,kw,ti.

9. 4 or 5 or 6 or 7 or 8

10. 3 and 9

11. $tumor.ab,kw,ti.

12. glio$ma.ab,kw,ti.

13. carcin$.ab,kw,ti.

14. $vestibul$.ab,kw,ti.

15. hearing.ab,kw,ti.

16. 10 not (11 or 12 or 13 or 14 or 15)

17. Hyperacute injury marker.ab,kw,ti.

18. hyperintense acute reperfusion marker.ab,kw,ti.

19. pcFLAIR.ab,kw,ti.

20. 16 or 17 or 18 or 19

21. limit 20 to review

22. 20 not 21

23. limit 22 to conference abstract

24. 22 not 23

25. limit 24 to human

26. limit 25 to english
