# Supplementary material for: CSF enhancement on post-contrast fluid-attenuated inversion recovery images; a systematic review
Source: Neuroimage Clin. 2020 Oct 2;28:102456. doi: 10.1016/j.nicl.2020.102456 (PMC7559862; doi:10.1016/j.nicl.2020.102456)
Supplement: Supplementary data 2 [file mmc2.pdf]

Supplemental table e-1: Study quality assessment according to Newcastle-Ottawa scale. (Herzog et al., 2013; Wells GA, 2017)

	Selection				Comparability		Outcome		
	Representativeness of the sample	Sample size ≥ 30	Non-respondents*	Ascertainment of exposure**	Study controls for time from contrast administration to image acquisition	Study controls for contrast agent dosage	Assessment of outcome (2 points if blinded to clinical data)	Statistical test***	Score total
**Stroke studies (mean score)**									5/8
**Acute ischemic stroke**									
Choi et al., 2017	1	1	n/a	2	1	1	2	n/a	8/8
Dechambre et al., 2000 ****	0	0	n/a	2	n/a	n/a	1	n/a	3/6
Forster et al., 2016	0	0	n/a	2	0	1	1	n/a	4/8
Gupta et al., 2017	0	1	n/a	1	1	1	2	n/a	6/8
Henning et al., 2008 ±****	0	0	n/a	2	n/a	n/a	1	n/a	3/6
Hjort et al., 2008	0	1	n/a	2	1	0	1	n/a	5/8
Kim et al., 2005	1	0	n/a	1	1	0	1	n/a	4/8
Latour et al., 2004 ±, Warach et al., 2004 ±	1	1	n/a	1	0	1	2	n/a	6/8
Luby et al., 2019	1	1	n/a	1	0	0	1	n/a	4/8
Nadareishvili et al., 2018	0	1	n/a	2	1	1	1	n/a	6/8

Ostwaldt et al., 2014 #	0	0	n/a	2	0	0	1	n/a	3/8
Ostwaldt et al., 2015 #	1	1	n/a	2	0	0	1	n/a	5/8
Villringer et al., 2017 #	0	1	n/a	1	0	1	1	n/a	4/8
**Acute ischemic stroke or transient ischemic attack**									
Lee et al., 2015 *	1	1	n/a	1	0	0	2	n/a	5/8
Lee et al., 2016 *	1	1	n/a	1	1	1	1	n/a	6/8
Lee et al., 2018	0	1	n/a	1	0	0	2	n/a	4/8
Rozanski et al., 2010 #	1	1	n/a	2	0	0	2	n/a	6/8
**Spontaneous intracerebral hemorrhage**									
Kidwell et al., 2011	0	1	n/a	1	0	0	2	n/a	4/8
Jolink et al., 2019	1	1	n/a	2	1	1	2	n/a	8/8
**Acute ischemic stroke, spontaneous intracerebral hemorrhage or transient ischemic attack (mixed cases)**									
Barr et al., 2010	1	1	n/a	2	1	1	2	n/a	8/8
**Post-cardiovascular and intracranial vascular surgery studies (mean score)**									5/8
**Treatment for carotid artery disease**									
Cho et al., 2014	0	1	n/a	2	1	0	2	n/a	6/8
Ogami et al., 2011	1	0	n/a	2	0	1	1	n/a	5/8
Wilkinson et al., 2000	0	0	n/a	2	1	1	1	n/a	5/8

**Aneurysm treatment**									
Li et al., 2018	1	1	n/a	1	0	1	1	n/a	5/8
Suthiphosuwan et al., 2018	1	1	n/a	1	1	1	1	n/a	6/8
**Cardiac surgery**									
Merino et al., 2013	0	1	n/a	1	0	1	1	n/a	4/8
**MS studies (mean score)**									7/8
Absinta et al., 2015	1	1	n/a	1	1	1	2	n/a	7/8
Bergsland et al., 2019 $	0	1	n/a	2	1	1	2	n/a	7/8
Coulette et al., 2019	1	1	n/a	1	1	1	2	n/a	7/8
Eisele et al., 2015	1	1	n/a	2	1	1	2	n/a	8/8
Harrison et al., 2017 &	0	0	n/a	2	1	1	2	n/a	6/8
Ighani et al., 2020 &	0	1	n/a	2	1	1	2	n/a	7/8
Jonas et al., 2018 &	0	1	n/a	2	1	1	2	n/a	7/8
Zivadinov et al., 2017 $	1	1	n/a	2	1	1	2	n/a	8/8
Zivadinov et al., 2018 $	1	1	n/a	2	1	1	2	n/a	8/8
Zurawski et al., 2020	0	1	n/a	2	1	1	2	n/a	7/8

**Studies in meningitis (mean score)**									5/8
Ahmad et al., 2005	0	1	n/a	2	1	1	2	n/a	7/8
Alonso et al., 2015	0	0	n/a	2	0	0	2	n/a	4/8
Fukuoka et al., 2010	0	0	n/a	2	1	1	1	n/a	5/8
Splendiani et al., 2005	0	0	n/a	2	1	1	1	n/a	5/8
**Studies in other diseases (mean score)**									6/8
**Memory clinic patients**									
Freeze et al., 2017 @	1	1	n/a	2	1	1	2	n/a	8/8
Freeze et al., 2019 @****	0	0	n/a	2	n/a	n/a	2	n/a	4/6
**Mixed (non-MS) infectious and non-infectious diseases**									
Absinta et al., 2017	1	1	n/a	1	1	1	1	n/a	6/8
**Familial amyloid polyneuropathy**									
Hirai et al., 2005	1	0	n/a	2	1	1	1	n/a	6/8

Abbreviations: n/a, not applicable; MS, multiple sclerosis.

±*#&$@ studies with (suspected) overlapping study samples.

*Because of inclusion bias due to the fact that participants need to be eligible for MRI and GBCA administration (i.e., cases with MRI contraindications such as cardiac pacemaker or cases with renal failure are typically excluded) this item was excluded from the study quality assessment scale.

** Studies that did not include a pre-contrast FLAIR scan for all participants received 1 point on this item, studies that included pre-contrast FLAIR scans for all participants received 2 points.

*** Because frequencies of CSF enhancement were the primary variable of interest in this review (and these do not require a statistical test within individual studies), this item was excluded from the study quality assessment scale.

**** These studies only selected cases with known CSF enhancement positivity.
