# Supplementary material for: CSF enhancement on post-contrast fluid-attenuated inversion recovery images; a systematic review
Source: Neuroimage Clin. 2020 Oct 2;28:102456. doi: 10.1016/j.nicl.2020.102456 (PMC7559862; doi:10.1016/j.nicl.2020.102456)
Supplement: Supplementary data 3 [file mmc3.pdf]

Supplemental table e-2: Population details and prevalence of cerebrospinal fluid enhancement.

Study	Design	Population details	Mean age(y %female	% with CS enhancement (n/N)
**Stroke studies**				
**Acute ischemic stroke**				
Choi et al., 2017	CS/R	Large artery atherosclerosis (n=75), small vessel occlusion (n=88), cardioembolism (n=31), strokes of other determined etiology (n=4), strokes with undetermined etiology (n=66)	69, 41%	25% (67/264)
Dechambre et al., 2000	CS/R	Acute ischemic stroke with HARM (n=5)	76, 40%	100% (5/5)***
Eisele et al., 2015 ^		Acute ischemic stroke with HARM (n=5)	64, 20%	100% (5/5)***
Forster et al., 2016	CS/unk	Posterior circulation infarction including posterior cerebral artery (n=13) and basilar artery (n=3) occlusion	68.5**, 44%	50% (8/16)
Gupta et al., 2017	CS/R	Mechanical thrombectomy of large vessel occlusion (n=35)	64, 43%	51% (18/35)

Henning et al., 2008 ±	CS/R	Acute ischemic stroke (n=10) with HARM	79, 50%	100% (10/10)***
Hjort et al., 2008	CS/R	Acute ischemic stroke with thrombolytic therapy (n=33)	68, unk	12% (4/33)
Kim et al., 2005	CS/P	Acute middle cerebral artery territory infarction with intra-arterial thrombolysis combined with IV tPA or intra-arterial urokinase treatment	62, 50%	57% (8/14)
Latour et al., 2004 ±, Warach et al., 2004 ±	CS/P	Acute ischemic stroke (n=144)	73, 48%	33% (47/144)
Luby et al., 2019	CS/unk	Acute anterior circulation infarction with endovascular therapy with (n=45) or without (n=35) standard IV tPA	65**, 64%	65% (52/80)
Nadareishvili et al., 2018	CS/R	Acute ischemic stroke without thrombolytic therapy or hemorrhagic transformation (n=33)	73, 61%	27% (9/33)
Ostwaldt et al., 2014 #	CS/unk	Acute ischemic stroke (n=18)	69**, 28%	44% (8/18)
Ostwaldt et al., 2015 #	CS/P	Large artery atherosclerosis (n=145), cardioembolism (n=125), small-vessel occlusion (n=68), other determined etiology (n=11), undetermined etiology (n=180)	71**, 39%	18% (97/529)
Villringer et al., 2017 #	CS/P	Acute ischemic stroke (n=30)	unk	23% (7/30)

**Acute ischemic stroke or transient ischemic attack (mixed)**				
Lee et al., 2015 *	CS/R	Acute ischemic stroke with (n=501) and without (n=77) diffusion abnormalities	59, 39%	12% (70/578)
Lee et al., 2016 *	CS/P	Acute ischemic stroke or TIA (n=218)	71, 33%	5.5% (12/218)
Lee et al., 2018	CS/R	Acute ischemic stroke or TIA with acute or subacute endovascular therapy (n=45)	unk	4.4% (2/45)
Rozanski et al., 2010 #	CS/unk	Old (≥80y) cases with acute ischemic stroke or TIA (n=47)	84**, 51%	40% (19/47)
**Spontaneous intracerebral hemorrhage**				
Jolink et al., 2019	CS/P	Spontaneous ICH (n=31)	60, 29%	55% (17/31)
Kidwell et al., 2011	CS/R	Spontaneous ICH with available pcT2wFLAIR imaging (n=46)	65, 60%	85% (39/46)
**Acute ischemic stroke, spontaneous intracerebral hemorrhage or transient ischemic attack (mixed cases)**				
Barr et al., 2010	CS/P	Acute ischemic cerebrovascular syndrome (n=33), ICH (n=6), stroke mimic (n=1), no stroke (n=1)	62, 44%	41% (17/41)
**Post-cardiovascular and intracranial vascular surgery studies**				

**Treatment for carotid artery disease**				
Cho et al., 2014	CS/P	Carotid stenting (n=16) or endarterectomy (n=29)	68, 22%	18% (8/45)
Ogami et al., 2011	CS/P	Carotid artery stenting (symptomatic (n=11) and asymptomatic (n=10)	74, 29%	52% (11/21 procedures in 19 cases)
Wilkinson et al., 2000	CS/unk	Symptomatic carotid artery stenting (n=12)	68, 50%	100% (12/12)
**Aneurysm treatment**				
Li et al., 2018	CS/P	Flow diversion therapy for unruptured intracranial aneurysm(s)	59, 88%	83% (34/41)
Suthiphosuwan et al., 2018	CS/R	Endovascular coiling of unruptured intracranial aneurysms	57, 69%	52% (32/62 examinations in 58 cases)
		**Cardiac surgery**		
Merino et al., 2013	CS/P	Cardiac surgery (off-pump coronary artery bypass grafting (CABG) (n=6), on-pump CABG (n=7), on-pump valvular surgery (n=6)	67, unk	47% (9/19)
**MS studies**				

Absinta et al., 2015	CS/P, subset L/P	Clinically isolated syndrome (n=10), relapsing remitting MS (n=171), secondary progressive MS (n=44), primary progressive MS (n=74)	48**, 59%	25% (74/299)
Bergsland et al., 2019 $	CS/R/P	Relapsing remitting MS or secondary progressive MS (n=342)	unk	17% (58/342)
Coulette et al., 2019 ^^	CS/L/R	Acute optic neuritis with MS (n=56) or clinically isolated syndrome (n=17)	38, 68%	8% (6/73)
Eisele et al., 2015	CS/R	Clinically isolated syndrome (n=18), relapsing remitting MS (n=79), secondary progressive MS (n=9), primary progressive MS (n=6)	38, 70%	1% (1/112)
Harrison et al., 2017 &	CS/P	Relapsing remitting MS (n=21), secondary progressive MS (n=4), primary progressive MS (n=4)	45.1, 52%	90% (26/29)

Ighani et al., 2020 &	CS/unk	Relapsing remitting MS (n=31), secondary progressive MS (n=5), primary progressive mS (n=5)	46, 66%	80% (33/41)
Jonas et al., 2018 &	L/P	Relapsing remitting MS (n=21), secondary progressive MS (n=7), primary progressive MS (n=4)	49**, 65%	100% (31/31)
Zivadinov et al., 2017 $	CS/R	Relapsing remitting MS (n=27), secondary progressive MS (n=23)	47, 80%	50% (25/50)
Zivadinov et al., 2018 $	CS/R	Relapsing remitting MS (n=212), secondary progressive MS (n=32), and clinically isolated syndrome (n=14)	49, 76%	15% (39/258)
Zurawski et al., 2020	CS/unk	Relapsing remitting MS (n=30)	44, 67%	66% (20/30)
**Studies in meningitis**				
Ahmad et al., 2005	CS/P	Tuberculous meningitis (n=15), pyogenic meningitis (n=6), viral meningitis (n=10)	48, 45%	90% (28/31)
Alonso et al., 2015	CS/R	Acute aseptic meningitis	35, 43%	50% (7/14)
Fukuoka et al., 2010	CS/P	Infectious meningitis (n=5), meningoencephalitis (n=3)	62, 25%	75% (6/8)
Splendiani et al., 2005	CS/unk	Infectious meningitis (n=12)	46, 67%	100% (12/12)

**Studies in other diseases**				
**Memory clinic patients**				
Freeze et al., 2017 @	CS/P, subset L/P	Mild cognitive impairment (n=33), Alzheimer's disease (n=15)	70, 30%	33% (16/48)
Freeze et al., 2019 @	L/P	Mild cognitive impairment (n=2) with CSF enhancement	81, 0%	100% (2/2)***
**Susac syndrome**				
Coulette et al., 2019	CS/L/R	Cases with a definite (n=8) or probable (n=1) diagnosis of Susac syndrome	38, 89%	56% (5/9)
**Familial amyloid polyneuropathy**				
Hirai et al., 2005	CS/P	Tyr114Cys mutation (n=3), Val30Met mutation (n=3)	40, 67%	50% (3/6)
**Inflammatory and immune-mediated neurologic diseases**				
Absinta et al., 2017	CS/P	Neuromyelitis optica spectrum disorder, immune-mediated encephalitis, immune-mediated cerebellar ataxia, systemic inflammatory diseases with white matter MRI abnormalities not suggestive for MS, and Susac syndrome (n=51)	43, 61%	35% (18/51)
**Noninflammatory neurologic diseases**				

Absinta et al., 2017	CS/P	Small vessel disease, migraine, neurodegenerative diseases, and compressive myelopathy (n=38)	50, 71%	8% (3/38)
**Human T-lymphotropic virus (HTLV) infection**				
Absinta et al., 2017	CS/P	HTLV-associated myelopathy/tropical spastic paraparesis (n=25), asymptomatic HTLV infection (n=13)	53, 66%	45% (17/38)
**Human immunodeficiency virus (HIV) infection**				
Absinta et al., 2017	CS/P	Cases with HIV infection on antiretroviral therapy	52, 41%	21% (13/61)

Abbreviations: N,sample size; n,number of cases with pcT2wFLAIR CSF enhancement; AIS, acute ischemic stroke; TIA, transient ischemic attack; ICH, intracerebral hemorrhage; CS, cross-sectional; L, longitudinal; R, retrospective; P, prospective; CSF, cerebrospinal fluid; unk, unknown; MS, multiple sclerosis.

#*±&$@ Studies with (suspected) overlapping study samples.

** Mean not available, median reported instead.

*** Because these studies only selected cases based on CSF enhancement positivity, these cases are excluded from the pooled prevalence estimates as depicted in figure 3.

^ Stroke cases were included as positive control in this study, with the main focus on MS.

^^ MS cases were included as controls in this study, with the main focus on Susac syndrome.
