# Supplementary material for: CSF enhancement on post-contrast fluid-attenuated inversion recovery images; a systematic review
Source: Neuroimage Clin. 2020 Oct 2;28:102456. doi: 10.1016/j.nicl.2020.102456 (PMC7559862; doi:10.1016/j.nicl.2020.102456)
Supplement: Supplementary data 4 [file mmc4.pdf]

Supplemental table e-3: Population details and prevalence of cerebrospinal fluid enhancement in control cases

Study	Design	Population details	Age(y), %female	% with CSF enhancement (n/N)
Absinta et al., 2015 [%’]	CS/P	9 healthy volunteers, 19 healthy first-degree relatives of MS cases, 9 healthy individual asymptomatically infected with human T-cell lymphotropic virus type I	42**, 48%	3% (1/37)
Absinta et al. 2017 [%’]	CS/P	66 healthy volunteers	42, 48%	8% (5/66)
Freeze et al., 2017 [@]	CS/P, supset L/P	26 older healthy volunteers	73, 46%	19% (5/26)
Freeze et al., 2019 [@]	L/P	4 older healthy volunteers with baseline CSF enhancement	74, 50%	100% (4/4)
Ighani et al., 2020 [&]	CS/unk	5 healthy volunteers	44, 20%	60% (3/5)

Harrison et al., 2017 &	CS/P	3 healthy volunteers	37, 33%	67% (2/3)
Zurawski et al., 2020	CS/unk	15 healthy controls	44, 67%	7% (1/15)

Abbreviations: N, sample size; n, number of cases with pcT2wFLAIR CSF enhancement; CS, cross-sectional; P, prospective; CSF, cerebrospinal fluid; unk, unknown.

@'& Studies with (suspected) overlapping study samples.

** Mean not available, median reported instead.
