# Supplementary material for: CSF enhancement on post-contrast fluid-attenuated inversion recovery images; a systematic review
Source: Neuroimage Clin. 2020 Oct 2;28:102456. doi: 10.1016/j.nicl.2020.102456 (PMC7559862; doi:10.1016/j.nicl.2020.102456)
Supplement: Supplementary data 5 [file mmc5.pdf]

Supplemental table e-4: Terminology, definition and appearance of post-contrast FLAIR CSF space enhancement.

Study	Terminology	Definition	Appearance
**Stroke studies**			
**Acute ischemic stroke**			
Choi et al., 2017	HARM	Cortical and sulcal contrast retention	Appearance: linear cortical or sulcal enhancement. Location: 82% in stroke vascular territory, 18% in multiple vascular territories ipsilateral or contralateral regardless of infarction
Dechambre et al., 2000	High signal from the CSF	High signal in subarachnoid space (higher signal intensity compared to cortex)	Appearance/location: diffuse bilateral (n=2); diffuse limited to stroke hemisphere (n=2); focal next to ischemic lesion (n=1); enhancement progressively decreased in extent and intensity and disappeared completely within 3 (n=2), and 6 (n=2) days.
Forster et al., 2016	HARM	Focal enhancement in the subarachnoid space and/or the ventricles	Appearance: focal in the subarachnoid space and/or the ventricles. Location: occipital sulci (n=7), ambient cistern (n=1), in all cases in the same vascular territory but remote from acute ischemic lesion.

Gupta et al., 2017	HARM	The presence of gadolinium in the sulci or parenchyma	Appearance and location not specified
Henning et al., 2008 ±	HARM	Positive signal enhancement; verification of enhancement in the CSF space (not parenchyma)	Location: within the vascular territory of the acute stroke, appearance not specified.
Hjort et al., 2008	HARM	Hyperintensity in the CSF	Appearance and location not specified; Enhancement was observed both 2h and 24h after thrombolytic therapy
Kim et al., 2005	Sulcal hyperintensity	Presence of sulcal hyperintensity	Appearance: sulcal hyperintensity. Location: focally near the infarcted area in all cases; disappeared within 3 days after symptom onset.
Latour et al., 2004 ±, Warach et al., 2004 ±	HARM	Delayed gadolinium enhancement of CSF space	Location/appearance: focally in the sulcal space in the vascular territory of the acute stroke (n=21), both focally and diffusely within the ventricles (n=20) and only diffusely in the ventricles (n=6), median time from onset of ischemia to observation of HARM was 13h; CSF enhancement was observed as late as 5 days after prior CA. HARM was not evident within 10min after CA.
Luby et al., 2019	HARM	Enhancement in the CSF spaces, especially in the sulci and on the cortical surface	HARM was rated on a semi-quantitative scale; none, present but not severe, severe occupying at least 10 slices but focal to the acute diffusion-weighted imaging ischemic

			lesion, or severe occupying at least 10 slices but diffuse and not just limited to the territory where the acute ischemic lesion was present.
Nadareishvili et al., 2018	HARM	Enhancement of the CSF space (sulci, ventricles, background, or vitreous appears hyperintense and continuous across >10 slices)	Appearance: 82.5% had enhancement occupying at least 10 slices. Location not specified.
Ostwaldt et al., 2014 #	HARM	CSF enhancement in the sulcal space in the affected vascular territory or within the ventricles	Appearance and location not specified
Ostwaldt et al., 2015 #	HARM	Hyperintensities in sulci or ventricles on >2 consecutive slices	Location: within the vascular territory of the acute stroke. HARM was present on all follow-up images in most cases (n=6), occurred after 3 doses of CA (28.5h after symptom onset) in 1 case, and appeared after 2 doses of CA (7.2h after symptom onset) and had disappeared 12 hours later (n=1)
Villringer et al., 2017 #	HARM	-	Appearance and location not specified
**Acute ischemic stroke or transient ischemic attack**			

Lee et al., 2015 *	HARM	Linear contrast enhancement (sulcal enhancement)	Appearance: focal linear contrast leakage. Location: Both cerebral sulci (n=4), both occipital sulci (n=2), cerebellar sulcus (n=1), both parieto-occipital sulci (n=1), frontal and occipital sulci (n=1), both high frontoparietal sulci (n=1). Focal enhancement became diffuse on <24h FU MRIs in all cases (n=5). Cases with >24h FU MRI showed a small amount of contrast leakage (n=1) or no leakage (n=5)
Lee et al., 2016 *	HARM	Linear contrast enhancement (abnormal cortical or sulcal enhancement)	Appearance: linear contrast enhancement along the cortical surface near acute infarctions. Location: multifocal (n=10, covering multiple vascular territories in 8 cases and in the same vascular territory as the infarction in 2 cases), focal in the same vascular territory as the acute infarction (n=1), and focal in the absence of infarction (n=1); initial linear enhancement spread diffusely to fill the subarachnoid space 2.5-19h later, and disappeared in the subset of cases who were again imaged 3-5 days later.
Lee et al., 2018	HARM	-	Appearance and location not specified
Rozanski et al., 2010 #	HARM	CSF enhancement in sulci and/or ventricles	Appearance and location not specified
**Spontaneous intracerebral hemorrhage**			
Kidwell et al., 2011	HARM	Hyperintense signal within the CSF spaces	Sulcal enhancement was noncontiguous with the hematoma in all cases. 50% showed moderate to severe HARM

Jolink et al., 2019	HARM	Hyperintense signal in normal-appearing brain or CSF, visually distinct and anatomically non-contiguous with the hematoma	Location: cortical (94%), deep and cortical (6%), never infratentorial; Both hemispheres (53%), symptomatic hemisphere (12%), contralateral hemisphere (35%). Appearance: 24% single punctate, 29% multi-punctate, 18% focal sulcal enhancement, 29% bilateral and diffuse; anatomically non-contiguous with the hematoma
**Acute ischemic stroke, spontaneous intracerebral hemorrhage or transient ischemic attack (mixed cases)**			
Barr et al., 2010	HARM	Enhancement of the CSF space (sulci, ventricles, background, or vitreous appears hyperintense and continuous across >10 slices)	Appearance and location not specified
**Post-cardiovascular and intracranial vascular surgery studies**			
**Treatment for carotid artery disease**			
Cho et al., 2014	HARM	Delayed gadolinium enhancement of the CSF space	Appearance and location not specified

Ogami et al., 2011	Enhancement of the CSF space	Hyperintensity or obscuration of the CSF space	Location: all CSF enhancements were distributed within the MCA territory at the core of the watershed area between the MCA and the PCA. Three lesions were recognized in the watershed area between the MCA and the ACA. 0% in the basal cistern
Wilkinson et al., 2000	LME	Abnormal accumulation of contrast media in the pia and/or arachnoid mater	Location: 50% unilateral enhancement of the leptomeninges primarily MCA territory, 50% focal enhancement adjacent to areas of old ischemic damage; 0% showed LME in ACA territory, LME in PCA territory was minimal; Ipsilateral to the stented carotid artery (n=11), contralateral to the stent in 1 case (the opposite carotid artery was occluded). In all cases LME became more prominent after 2nd CA.
**Aneurysm treatment**			
Li et al., 2018	LME	Leptomeningeal enhancement	38% focal, 47% lobar, 15% hemispheric (based on rater 1)
Suthiphosuwan et al., 2018	CSHF (cortical and sulcal hyperintensity on gadolinium-enhanced FLAIR)	Cortical or sulcal hyperintensity equal to or greater than a superficial cortical vein	Location: right ACA territory (47%), right MCA territory (56%), left ACA territory (13%), left MCA territory (13%), right PCA territory (19%), left PCA territory (13%), left cerebellar hemispheric territory (6%); enhancement was observed in the arterial territories exposed to iodinated contrast media during angiography.
**Cardiac surgery**			

Merino et al., 2013	HARM	Enhancement of the CSF	HARM was present as mild/moderate background signal (n=4), sulcal(severe)+background (severe)+ventricular(mild/moderate) (n=1), sulcal(severe)+background (mild/moderate) (n=1) or only sulcal(mild/moderate) (n=3)
**Multiple sclerosis studies**			
Absinta et al., 2015	LME	Signal intensity within the subarachnoid space substantially greater than that of brain parenchyma	109 foci were detected in 74 cases. Location: 19% cerebral convexity 56% within a sulcus, 17% along a dural fissure, 7% traversing several of these areas (8/109, 7%). In one case high resolution T2* images were acquired at 7T MRI, which showed that LME was perivascular. Of 62 foci that were measured at FU imaging, 53 were stable in shape and size, 1 disappeared, 2 fluctuated over time, and 6 new foci were detected in 4 cases. 1 control case presented with a single focus of LME in the right frontal region.
Bergsland et al., 2019 $	LMCE	Signal intensity within the subarachnoid space that was substantially greater than that of brain parenchyma	80 foci detected in 58 cases. Appearance: 65% nodular, 20% plate-like, 15% linear.
Eisele et al., 2015	Cortical or leptomeningeal contrast enhancement	Sulcal or leptomeningeal hyperintensity	In one case focal CSF enhancement was observed in the right temporal lobe.

Harrison et al., 2017 &	LME	Hyperintensities on subtraction images within the leptomeningeal space (pia, subarachnoid, or subarachnoid space)	Median of 2 foci per case (range 0-8). Appearance: nodular foci in 51% of cases; spread/fill foci in 76% of cases; control cases (n=2) had two or three nodular foci.
Ighani et al., 2020 &	LME	Hyperintensities on pcFLAIR images and subtraction images within the leptomeningeal space	Median of 3 foci per case (range 0-14). Appearance: spread/fill-gyral foci in 61% of cases, spread/fill-sulcal foci in 59%, nodular in 32%. Locaton: infratentorial in 10% of cases
Jonas et al., 2018 &	Meningeal enhancement	Hyperintensities in the meningeal space	284 foci detected in 31 cases. Appearance: 40% subarachnoid spread/fill foci, 5% subarachnoid nodular foci, 38% vessel wall pattern, 16% dural nodular foci; Location: 44% frontal, 31% parietal, 15% occipital, 8% temporal lobe, 1% cerebellar. At 1-year FU, persistence was noted in 89% overall foci, and in 82% at 2-year FU.
Zivadinov et al., 2017 $	LMCE	Signal intensity within the subarachnoid space that was substantially greater than that of brain parenchyma	61 foci detected in 25 cases. Appearance: 80% nodular, 13% plate-like, 7% linear. Location: 21% in sulci, 79% cortical convexity; 41% right hemisphere, 59% left hemisphere; 93% supratentorial, 7% infratentorial; lobar foci: 20% frontal, 18% parietal, 16% temporal, 39% occipital lobe.

Zivadinov et al., 2018 $	LMCE	Signal intensity within the subarachnoid space that was substantially greater than that of brain parenchyma	61 foci were detected in 39 cases using a subtraction method. Appearance: 68% nodular, 11% plate-like, 20% linear. Location: 70% sulci, 30% cortical convexity; 64% right hemisphere, 34% left hemisphere, all supratentorial. 30% frontal, 36% parietal, 8% temporal, and 25% occipital lobe.
Zurawski et al., 2020	LME	Hyperintense signal on pcFLAIR with no signal on pre-contrast FLAIR	54 foci were identified in 20 cases. Appearance: all cases had at least 1 nodular LME focus, 8 cases also had spread/fill LME. Location: 43% left hemisphere, 57% right hemisphere; 52% frontal, 31% parietal, 13% temporal, 4% occipital.
**Studies in meningitis**			
Ahmad et al., 2005	LME	Thick, long or nodular meningeal enhancement, noted on >3 contiguous images, and extending deep into sulcal bases	Appearance and location not specified
Alonso et al., 2015	LME	Leptomeningeal and/or sulcal contrast enhancement	Distinct leptomeningeal enhancement along each cerebral lobe of both hemispheres and the cerebellum (n=4); subtle contrast enhancement confined to the supratentorial leptomeninges (n=3).

Fukuoka et al., 2010	LME	Abnormal leptomeningeal enhancement	Abnormal leptomeningeal enhancement in the sulci.
Splendiani et al., 2005	Pathological leptomeningeal altered signals	Abnormal enhancement in sulci, cisterns, ventricles, or any combination of these	Abnormal enhancement was marked and circumscribed or diffuse.
**Studies in other diseases**			
**Memory clinic patients**			
Freeze et al., 2017 @	Pericortical enhancement	Hyperintense signal substantially brighter than the proximal parenchymal signal intensity, occurring in the subarachnoid space	32 pericortical foci were detected in 21 cases (including control cases). Location: 31% PCA, 28% MCA, 41% ACA; 47% left hemisphere, 53% right hemisphere; 38% on the cortical convexity, 38% within a sulcus, 3% traversed several sulci, 22% within the longitudinal fissure. 1 focus in 23 cases, 2 foci in five cases, 3 foci in three cases. In addition, 1 focus was found in a perivascular space. At FU all foci remained stable over time.

Freeze et al., 2019 @	Pericortical enhancement	Hyperintense signal substantially brighter than the proximal parenchymal signal intensity, occurring in the subarachnoid space	All pericortical foci had remained stable since baseline, one new leakage focus was detected. Foci appeared visually more intense on heavily T2w pcT2wFLAIR images compared to regular pcT2wFLAIR images. In one case, widespread signal enhancement within the sulci became visible on the heavily T2w pcT2wFLAIR scan several hours after CA, which was not present on the scan shortly after CA.
**Susac syndrome**			
Coulette et al., 2019	LME	Hyperintensity within the subarachnoid space substantially greater than that of the brain parenchyma	All cases with Susac syndrome had at least 3 LME lesions of which at least one in the posterior fossa. MS cases never had more than 2 lesions and LME was never detected within the posterior fossa. LME appeared as punctate, linear, or mixed. On FU MRIs (n=53) LME was seen in 7 of 9 cases with Susac syndrome on 40 follow-up MRIs. LME lesion load remained unchanged in 36%, was decreased in 23%, and was increased in 41% of MRIs.
**Familial amyloid polyneuropathy**			
Hirai et al., 2005	CSF enhancement	Contrast enhancement of the leptomeninges	LME was seen immediately after CA especially in the superior cerebellar cisterns, Sylvian fissures, interhemispheric fissures, and cerebral sulci. 3 and 6 hours after CA there was marked and diffuse CSF enhancement. At 24 hours there was mild CSF enhancement in the sulci.

Mixed (non-MS) infectious and non-infectious diseases

Absinta et al., 2017	LME	Signal intensity within the subarachnoid space substantially greater than that of brain parenchyma	99 foci were detected in 56 cases, 75% had a single focus and 25% had multiple foci. Appearance: linear or nodular. Location: mostly supratentorial.

#*±&$@ Studies with (suspected) overlapping study samples.

Abbreviations: CSF, cerebrospinal fluid; FU, follow-up; HARM, Hyperintense acute reperfusion (injury) marker; LME, leptomeningeal enhancement; LMCE, leptomeningeal contrast enhancement; FLAIR, fluid-attenuated inversion recovery

; -, not available.
