# Supplementary material for: CSF enhancement on post-contrast fluid-attenuated inversion recovery images; a systematic review
Source: Neuroimage Clin. 2020 Oct 2;28:102456. doi: 10.1016/j.nicl.2020.102456 (PMC7559862; doi:10.1016/j.nicl.2020.102456)
Supplement: Supplementary data 6 [file mmc6.pdf]

Supplemental table e-5: Post-contrast FLAIR acquisition details.

Study	Pre-contrast FLAIR	Field strength	Contrast type	dose	Time from CA to acquisition	TR	TI	TE	Slice thickness, 2D/3D
**Stroke studies**									
**Acute ischemic stroke**									
Choi et al., 2017	Yes	3T	-	0.2mmol/kg	5min	11000	-	125	5mm, 2D
Dechambre et al., 2000	Yes	1.5T	Gd-DTPA	0.1mmol/kg	18-42h, 3d, 6d	10002	2200	148	5mm, 2D
Forster et al., 2016	Yes	1.5T	Gd-DOTA	0.1mmol/kg	Within 24h	8500/9000	2400/2500	115/89	5mm, 2D
Gupta et al., 2017	No	1.5T	-	0.1mmol/kg	24h	11000	-	140	5mm, 2D
Henning et al., 2008 ±	Yes	1.5T	-	-	-	9000	1750	85	7mm, 2D
Hjort et al., 2008	Yes	1.5T or 3T	Gd-BT-Do3A	-	2h and 24h	8650	-	120	-

Kim et al., 2005	-	1.5T	Gd-DTPA	20ml, unclear if there were multiple CAs	~2h, 3d	10000	2200	133	5mm, 2D
Latour et al., 2004 ±, Warach et al., 2004 ±	Yes, in all but 5 cases	1.5T	Gd-DTPA	0.1mmol/kg	10 min + variable depending on clinical needs, up to 5d	-	-	-	-
Luby et al., 2019	-	3T	Gd-DTPA	0.1mmol/kg, variable number of doses	-	-	-	-	-
Nadareishvili et al., 2018	Yes	1.5T	Gd-DTPA	0.1mmol/kg	24h	-	-	-	-
Ostwaldt et al., 2014 #	Yes	3T	Gd-BT-Do3A	5ml, multiple CAs	1.5-5h + multiple examinations	8000	-	100	5mm, 2D
Ostwaldt et al., 2015 #	Yes	3T	Gd-BT-Do3A	Between 0.04 and 0.31mmol/kg	~24h + variable depending on clinical needs	8000	-	100	5mm, 2D

Villringer et al., 2017 #	-	3T	Gd-BT-Do3A	10ml	-	-	-	-	-
**Acute ischemic stroke or transient ischemic attack (mixed cases)**									
Lee et al., 2015 *	No	1.5T or 3T	Gd-BT-Do3A	0.2mmol/kg or fixed dose of 15ml	~5 min + multiple examinations	11000	-	120-140	5mm, 2D
Lee et al., 2016 *	No	1.5T or 3T	Gd-BT-Do3A	15ml	~5min +2.5-19h + 3-5d	11000	2500	120-140	5mm, 2D
Lee et al., 2018	-	-	-	-	-	-	-	-	-
Rozanski et al., 2010 #	Yes	3T	Gd-BT-Do3A	4-6ml	~24h	8000	-	100	5mm, 2D
Jolink et al., 2019	Yes	7T	Gd-BT-Do3A or Gd-DOTA	0.1mmol/kg (max 10ml or 30ml)	>10min	8000	2325	300	<1mm, 3D
**Spontaneous intracerebral hemorrhage**									

Kidwell et al., 2011	Yes, at least in 78%	1.5T or 3T	-	Variable doses (1-4)	-	-	-	-	-
**Acute ischemic stroke, spontaneous intracerebral hemorrhage or transient ischemic attack (mixed cases)**									
Barr et al., 2010	Yes	3T	Gd-DTPA	0.1mmol/kg	~24h	-	-	-	-
**Post-cardiovascular and intracranial vascular surgery studies**									
**Carotid artery disease treatment**									
Cho et al., 2014	Yes	1.5T	-	-	~24h	10002	2200	97.5	-, 2D
Ogami et al., 2011	Yes	1.5T	Gd-DTPA	15ml	On average >19h	8000	2000	114	6mm, 2D
Wilkinson et al., 2000	Yes	1.5T	Gd-DTPA	2x 20ml	Short (within the same session as CA)	6000	1800	95.9	5mm, 2D
**Aneurysm treatment**									

Li et al., 2018	Yes, in 81% of cases	1.5T or 3T	Gd-BOPTA	0.1mmol/kg	-	6000	1700	135	1.6mm, 3D
Suthiphosuwan et al., 2018	No	1.5T	Gd-BOPTA	20ml	Short (after contrast-enhanced MRA)	11000	2800	140	5mm, 2D
**Cardiac surgery**									
Merino et al., 2013	Yes, in ~50% of cases	1.5T or 3T	-	0.1mmol/kg	<1h in ~ 50% of patients, 30.4h in other half	9000	2200/2600	140-120	3.5-4mm, 2D
**MS studies**									
Absinta et al., 2015	Yes, in 46% of cases	3T	Gd-DTPA (15%) or Gd-BT-Do3A (85%)	0.1mmol/kg	≥10min; median (IQR) MS cases = 26 (11) min	4800/6000	1800/1860	125/354	1mm, 3D

Bergsland et al., 2019 $	Yes	3T	Gd-DTPA	0.1mmol/kg	10min	9000	2420	600 (TEeff = 110)	1mm, 3D
Coulette et al., 2019	No	3T	-	0.1mm/kg	12min	8000	2400	388	1mm, 3D
Eisele et al., 2015	Yes	3T	Gd-DOTA	0.1mmol/kg	>10min	8500	2500	136	5mm, 2D
Harrison et al., 2017 &	Yes	7T	Gd-HP-Do3A	0.1mmol/kg	20min	8000	2077	400	0.7mm, 3D
Ighani et al., 2020 &	Yes	7T	Gd-HP-Do3A	0.1mmol/kg	20min	8000	2077	400	0.7mm, 3D
Jonas et al., 2018 &	Yes	7T	Gd-HP-Do3A	0.1mmol/kg	20min	8000	2077	400	0.7mm, 3D
Zivadinov et al., 2017 $	Yes	3T	Gd- BT-DO3A	0.1mmol/kg	10min	-	-	-	1mm, 3D; -, 2D
Zivadinov et al., 2018 $	Yes	3T	Gd-DTPA	0.1mmol/kg	10min	9000	2420	600 (TEeff = 110)	1mm, 3D
Zurawski et al., 2020	Yes	7T	Gd-DOTA	0.1mmol/kg	10min	9000	2500	301	0.7mm, 3D

**Studies in meningitis**									
Ahmad et al., 2005	Yes	1.5T	Gd-DTPA-BMA	0.1mm/kg	3-4min	9000	2500	TEeff = 88	5mm, 2D
Alonso et al., 2015	Yes	3T	-	-	-	-	-	-	-
Fukuoka et al., 2010	Yes	3T	Gd-DTPA	0.1mmol/kg	Short (1 or 2 sequences after CA)	6000	2000	420	0.9mm, 3D
Splendiani et al., 2005	Yes	1.5T	-	0.1mmol/kg	0-2min	10000	2200	147	5mm, 2D
**Studies in other diseases**									
**Memory clinic patients**									
Freeze et al., 2017 @	Yes	3T	Gd-BT-DO3A	0.1mmol/kg, max 10ml	16min	4800	1650	290	1mm, 3D
Freeze et al., 2019 @	Yes	3T	Gd-BT-DO3A	0.1mmol/kg	16min and >1.5h	4800	1650	290 and 500	1mm, 3D
**Mixed (non-MS) infectious and non-infectious diseases**									

Absinta et al., 2017	Yes, in 81% of cases	3T	-	0.1mmol/kg	≥10min	-	-	-	-
**Familial amyloid polyneuropathy**									
Hirai et al., 2005	Yes	1.5T	Gd-DTPA	0.1mmol/kg	0min and 3h, 6h, and 24h	6000	2000	120	5mm, 2D

Abbreviations: TR, repetition time; TI, inversion time; TE, echo time; -, unknown; ~, approximately; Gd-DTPA, gadopentetate (Magnevist ®); Gd-DOTA, gadoterate (Dotarem ®); Gd-BT-Do3A, gadobutrol (Gadavist ®); Gd-BOPTA, gadobenate (MultiHance ®); MRA, magnetic resonance angiography; IQR, interquartile range; MS, multiple sclerosis; Gd-HP-Do3A, gadoteridol (Prohance ®); Gd-DTPA-BMA, gadodiamide (Omniscan ™); TEeff, effective echo time.

#*±&$@ Studies with (suspected) overlapping study samples.
