# Supplementary material for: CSF enhancement on post-contrast fluid-attenuated inversion recovery images; a systematic review
Source: Neuroimage Clin. 2020 Oct 2;28:102456. doi: 10.1016/j.nicl.2020.102456 (PMC7559862; doi:10.1016/j.nicl.2020.102456)
Supplement: Supplementary data 7 [file mmc7.pdf]

Supplemental table e-6: Overview of studies that examined potential risk factors and conventional imaging markers of cerebrospinal fluid enhancement.

	Possible risk factors						Conventional imaging markers			
	Age	Sex	HyT	DM	HL	Sm	WMH	LI	CMB	Atrophy
**Stroke studies**										
**Acute ischemic stroke**										
Choi et al., 2017	No	No	No	No	No	No	-	-	-	-
Dechambre et al., 2000	-	-	-	-	-	-	-	-	-	-
Forster et al., 2016	No	No	-	-	-	-	-	-	-	-
Gupta et al., 2017	No	No	No	Yes (-)	No	-	-	-	-	-
Henning et al., 2008	-	-	-	-	-	-	-	-	-	-

Hjort et al., 2008	-	-	-	-	-	-	-	-	-	-
Kim et al., 2005	-	-	-	-	-	-	-	-	-	-
Latour et al., 2004 , Warach et al., 2004	Yes (+)	No	No	-	-	-	-	-	-	-
Luby et al., 2019	-	-	-	-	-	-	-	-	-	-
Nadareishvili et al., 2018	No	No	No	No	-	-	-	-	-	-
Ostwaldt et al., 2014 #	No	No	No	No	No	No	-	-	-	-
Ostwaldt et al., 2015 #	Yes (+)	-	-	-	-	-	-	-	-	-
Villringer et al., 2017 #	-	-	-	-	-	-	-	-	-	-
**Acute ischemic stroke or transient ischemic attack (mixed cases)**										
Lee et al., 2015 *	Yes (+)	No	No	No	No	No	-	-	-	-

Lee et al., 2016 *	-	-	-	-	-	-	-	-	-	-
Lee et al., 2018	-	-	-	-	-	-	-	-	-	-
Rozanski et al., 2010 #	Yes (+)	No	No	No	Yes (-)	-	No	-	-	-
**Spontaneous intracerebral hemorrhage**										
Kidwell et al., 2011	-	-	-	-	-	-	-	-	-	-
Jolink et al., 2019	No	No	No	No	-	No	No	-	Yes (+)	-
**Acute ischemic stroke, spontaneous intracerebral hemorrhage or transient ischemic attack (mixed cases)**										
Barr et al., 2010	Yes (+)	No	No	-	-	-	-	-	-	-
**Post-cardiovascular and intracranial vascular surgery studies**										
**Carotid artery disease treatment**										
Cho et al., 2014	Yes (+)	No	No	No	No	No	No	-	-	-
Ogami et al., 2011	Yes (+)	No	No	No	No	-	-	-	-	-

Wilkinson et al., 2000	-	-	-	-	-	-	-	-	-	-
**Aneurysm treatment**										
Li et al., 2018	No	-	No	No	-	No	-	-	-	-
Suthiphosuwan et al., 2018	No	No	-	-	-	-	-	-	-	-
**Cardiac surgery**										
Merino et al., 2013	-	-	-	-	-	-	-	-	-	-
**Studies in Multiple Sclerosis**										
Absinta et al., 2015	Yes (+)	No	-	-	-	-	No	-	-	Yes (+)
Bergsland et al., 2019 $	-	-	-	-	-	-	-	-	-	Yes (+)
Coulette et al., 2019	-	-	-	-	-	-	-	-	-	-

Eisele et al., 2015	-	-	-	-	-	-	-	-	-	-
Harrison et al., 2017 &	-	-	-	-	-	-	-	-	-	-
Ighani et al., 2020 &	-	-	-	-	-	-	No	-	-	Yes (+)
Jonas et al., 2018 &	-	-	-	-	-	-	-	-	-	-
Zivadinov et al., 2017 $	No	No	-	-	-	-	-	-	-	Yes (+)
Zivadinov et al., 2018 $	Yes (+), in relapsing remitting MS	No	-	-	-	-	-	-	-	-
Zurawski et al., 2020	No	No	-	-	-	-	Yes (+)	-	-	No
**Studies in meningitis**										

Ahmad et al., 2005	-	-	-	-	-	-	-	-	-	-
Alonso et al., 2015	-	-	-	-	-	-	-	-	-	-
Fukuoka et al., 2010	-	-	-	-	-	-	-	-	-	-
Splendiani et al., 2005	-	-	-	-	-	-	-	-	-	-
**Studies in other diseases**										
**Memory clinic patients**										
Freeze et al., 2017 @	Yes (+)	No	-	-	-	-	No	No	No	No
Freeze et al., 2019 @	-	-	-	-	-	-	-	-	-	-
**Mixed (non-MS) infectious and non-infectious diseases**										
Absinta et al., 2017	Yes (+)	No	-	-	-	-	-	-	-	No

**Familial amyloid polyneuropathy**													
Hirai et al., 2005	-	-	-	-	-	-				-	-	-	-

Abbreviations: HyT, hypertension; DM, diabetes Mellitus; H, hyperlipidemia; Sm, smoking; WMH, white matter hyperintensities; LI, lacunar infarcts; CMB, cerebral microbleeds; -, negative association; +, positive association.

#*±&$@% Studies with (suspected) overlapping study samples.
